# Evaluation of hemostasis in patients with end-stage renal disease

**DOI:** 10.1371/journal.pone.0212237

**Published:** 2019-02-20

**Authors:** Anja Gäckler, Hana Rohn, Ton Lisman, Tamas Benkö, Oliver Witzke, Andreas Kribben, Fuat H. Saner

**Affiliations:** 1 Department of Nephrology, University Hospital Essen, University Duisburg-Essen, Essen, Germany; 2 Department of Infectious Diseases, University Hospital Essen, University Duisburg-Essen, Essen, Germany; 3 Surgical Research Laboratory and Section of Hepatobiliary Surgery and Liver Transplantation, Department of Surgery, University of Groningen University Medical Center Groningen, Groningen, The Netherlands; 4 Department of General, Visceral and Transplant Surgery, University Hospital Essen, University Duisburg-Essen, Essen, Germany; Institut d'Investigacions Biomediques de Barcelona, SPAIN

## Abstract

An increased bleeding risk is reported for patients with end-stage renal disease. This study aims to analyze, whether bleeding risk can be assessed by global tests of hemostasis. Standard laboratory tests and an extended evaluation of hemostasis by rotational thromboelastometry, platelet function analyzer (PFA) and multiple electrode aggregometry as well as thrombin generation assays and measurement of fibrinolytic potential were performed in 20 patients on hemodialysis, 10 patients on peritoneal dialysis, 10 patients with chronic kidney disease stage G5 (CKD5) and in 10 healthy controls (HC). Hemoglobin was significantly lower in patients with end-stage renal disease versus HC (each p<0.01). Patients on peritoneal dialysis showed increased fibrinogen levels compared to HC (p<0.01), which were also reflected by FIBTEM results (each p<0.05). 41% of hemodialysis patients and 44% of CKD5 patients presented with prolonged PFA-ADP-test (p<0.05), while no patient on peritoneal dialysis and no HC offered this modification. Thrombin generating potential was significantly lower in patients on hemodialysis, while clot lysis time revealed a hypofibrinolytic state in patients on hemo- and peritoneal dialysis compared to HC (p<0.001). In conclusion, patients with end-stage renal disease have complex hemostatic changes with both hyper- and hypocoagulable features, which are dependent on use and type of dialysis. Hypercoagulable features include elevated fibrinogen levels and a hypofibrinolytic state, whereas hypocoagulable features include decreased thrombin generating capacity and platelet dysfunction. Our results may contribute to a more rational approach to hemostatic management in these patients.

## Introduction

An increased bleeding risk has been described for patients with end-stage renal disease. Bleeding occurs in about 50% of patients with end-stage renal disease [1, 2] reaching from minor events such as bruises and bleeding at venipuncture sites to menorrhagia, gastrointestinal blood loss, severe perioperative bleeding and retroperitoneal as well as intracranial hemorrhage. Bleeding can significantly contribute to mortality and morbidity and blood transfusions can lead to alloimmunization and thereby limit options for transplantation [3].

Bleeding diathesis is multifactorial and attributed to morphological changes of the vessels, anaemia, thrombocytopenia and uraemic disturbance of platelet adhesion and aggregation, coagulation and fibrinolysis [2, 4, 5].

Although predisposition to bleeding seems obvious, recommendations for risk determination are missing as no single factor or test has been established to detect individual bleeding risk in patients with end-stage renal disease.

On the other hand, the risk of venous thromboembolism is also increased in patients with end-stage renal disease [6, 7], making risk management even more challenging.

In addition, patients with end-stage renal disease do not represent a homogenous cohort as they include patients with pharmaceutical treatment only, patients with hemodialysis and patients with peritoneal dialysis. Comparative data including less frequently used tests such as rotational thromboelastometry, multiple electrode aggregometry, thrombin generation assays and test of fibrinolytic potential for determination and characterization of plasmatic coagulation and platelet function in those patients are rare. Therefore, this study aims to analyze, whether the bleeding risk in different subgroups of patients with end-stage renal disease can be objectified by extended evaluation of hemostasis using various global assays.

## Materials and methods

### Patient cohort and study design

Between August 2014 and April 2016 we enrolled a total of 50 participants in our study: 20 patients on hemodialysis (HD), 10 patients on peritoneal dialysis (PD), 10 patients with chronic kidney disease stage G5 (CKD5) as well as 10 healthy controls (HC). All participants showed no signs of acute illness, were non-smokers and had no known hemostatic disorder. Medication was recorded with regard to platelet function inhibitors and anticoagulation therapy.

Standard laboratory values (serum creatinine (sCrea), blood urea nitrogen (BUN), blood count, prothrombin time (PT), activated partial thrombin time (aPTT), international normalized ratio (INR), fibrinogen) and an extended evaluation of hemostasis by means of rotational thromboelastometry (ROTEM) as well as of platelet function by platelet function analyzer (PFA) and multiple electrode aggregometry (Multiplate) were performed. In addition, thrombin generation assays (TGA) and fibrinolytic potiential, represented by clot lysis time (CLT) were measured.

The study was performed in accordance with the Declaration of Helsinki and the International Conference on Harmonization Good Clinical Practice guidelines. All participants gave written informed consent for analysis of blood values and clinical data. The study was approved by the local ethics committee of the University of Duisburg-Essen (14-5755-BO).

### Blood sampling and measurements

Standard laboratory values were gathered from a routine blood drawing after clean venipuncture with additional extraction of a 3 ml trisodiumcitrate monovette and a 2 ml lithium heparin blood gas monovette (Sarstedt, Nümbrecht, Germany) for extended evaluation. In HD patients blood was drawn immediately before a dialysis session with an interval of at least 18 hours since the last treatment.

All laboratory measurements, except rotational thromboelastometry, multiple electrode aggregometry, TGA and fibrinolytic potential were performed by the central laboratory unit of the University Hospital Essen as part of the clinical routine.

The platelet function analyzer-100 (PFA) test measures platelet aggregate formation under high shear rates and with addition of either collagen/epinephrine or collagen/adenosine diphosphate (ADP) as agonists (PFA-Epi and PFA-ADP). Patients with a hematocrit <0.30 were excluded from measurement of PFA.

Thromboelastometry analysis was performed with a ROTEM delta system (Tem Innovations, Munich, Germany) according to the manufacturer´s instructions. The tests included EXTEM, INTEM, FIBTEM and APTEM.

As described previously, for EXTEM, 300 μl citrated whole blood was mixed with 20 μl tissue factor and 20 μl CaCl_2_ 0.2 mol/l. INTEM was performed with 300 μl citrated whole blood, 20 μl ellagic acid and 20 μl CaCl_2_ 0.2 mol/l. FIBTEM was performed as EXTEM with the addition of cytochalasin D for inhibition of platelets, whereas APTEM was performed with addition of tranexamic acid for inhibition of fibrinolysis [8].

Parameters assessed included clotting time (CT), clot firmness 10 minutes after CT (A10), maximum clot firmness (MCF) and maximum lysis (ML). All tests were performed for at least one hour.

Multiple electrode aggregomety was performed with a Multiplate analyzer (Roche Diagnostics, Mannheim, Germany) according to the manufacturer´s instructions. The tests included ADP, ASPI, TRAP and Risto low.

300 μl 0.9% normal saline solution was mixed with 300 μl of lithium heparin blood sample and incubated at 37°C for 3 min. ADPtest was perfomed with addition of 20 μl of ADP (6.5 μM). ASPItest was performed with addition of 20 μl of arachidonic acid (0.5 mM). TRAPtest was performed with addition of 20 μl of thrombin receptor-activating peptide 6 (32 μM) and Risto lowtest was performed with addition of 12 μl ristocetin (0.2 mg/ml). Aggregation was monitored by means of impedance over 6 min and is quantified by the area under the curve (AUC).

All samples were analyzed between 30–90 min post-drawing.

For measurement of TGA and fibrinolytic potential citrated whole blood was centrifuged at 3000 U/min for 15 min within 60 min post-drawing. Plasma was transferred to a different tube and centrifuged again at 3000 U/min for 20 min. The platelet-poor plasma was transferred again and stored at -80°C until measurement.

TGA were performed with the fluorimetric method described by Hemker, Calibrated Automated Thrombography according to the instructions of the manufacturer [9]. As described previously [10], coagulation was activated using a commercial trigger composed of recombinant tissue factor at a concentration of 5 pM and phospholipids at a concentration of 4 μM (Thrombinoscope BV, Maastricht, The Netherlands) in the presence of a soluble form of thrombomodulin. The lagtime, endogenous thrombin potential (ETP), peak height, and velocity index were derived from the thrombin generation curves by the Thrombinoscope software.

Fibrinolytic potential was assessed as described before [10] using a plasma-based lysis assay. Lysis of a tissue factor-induced clot exogenous tissue plasminogen activator (tPA) was determined by monitoring changes in turbidity during clot formation and subsequent lysis. CLT were derived from the clot-lysis turbidity profiles using in house-generated software. CLT was defined as the time from the midpoint of the clear to maximum turbid transition, representing clot formation, to the midpoint of the maximum turbid to clear transition, representing the lysis of the clot.

### Statistical analysis

Data are expressed as mean value ± standard error of the mean, if not declared otherwise. Comparisons among multiple groups were performed using Kruskal-Wallis test followed by Dunn´s post-hoc analysis. Additionally, the contribution of different types of renal insufficiency to exceedance of threshold values was evaluated by Fisher´s exact test. A p < 0.05 was considered significant.

## Results

A total of 50 patients were enrolled in the current study: 20 patients on hemodialysis (HD), 10 patients on peritoneal dialysis (PD), 10 patients with chronic kidney disease stage G5 (CKD5) as well as 10 healthy controls (HC). All participants showed no signs of acute illness and were non-smokers.

Mean age was 45.9 years (minimum 18 years; maximum 77 years) with no significant differences between groups. 50% of study participants were women.

Three patients within the HD group and one patient in the CKD5 group were on treatment with phenprocoumon, while nine patients on HD and 3 patients on PD were treated with aspirin. Patients treated with aspirin were excluded for interpretation of PFA-Epi and ASPItest. Patients treated with phenprocoumon were excluded for interpretation of INR, aPTT and thromboelastometry. No further platelet function inhibitors or anticoagulation therapies were used in the study cohort.

### Serum creatinine and blood urea nitrogen

**Fig 1:**
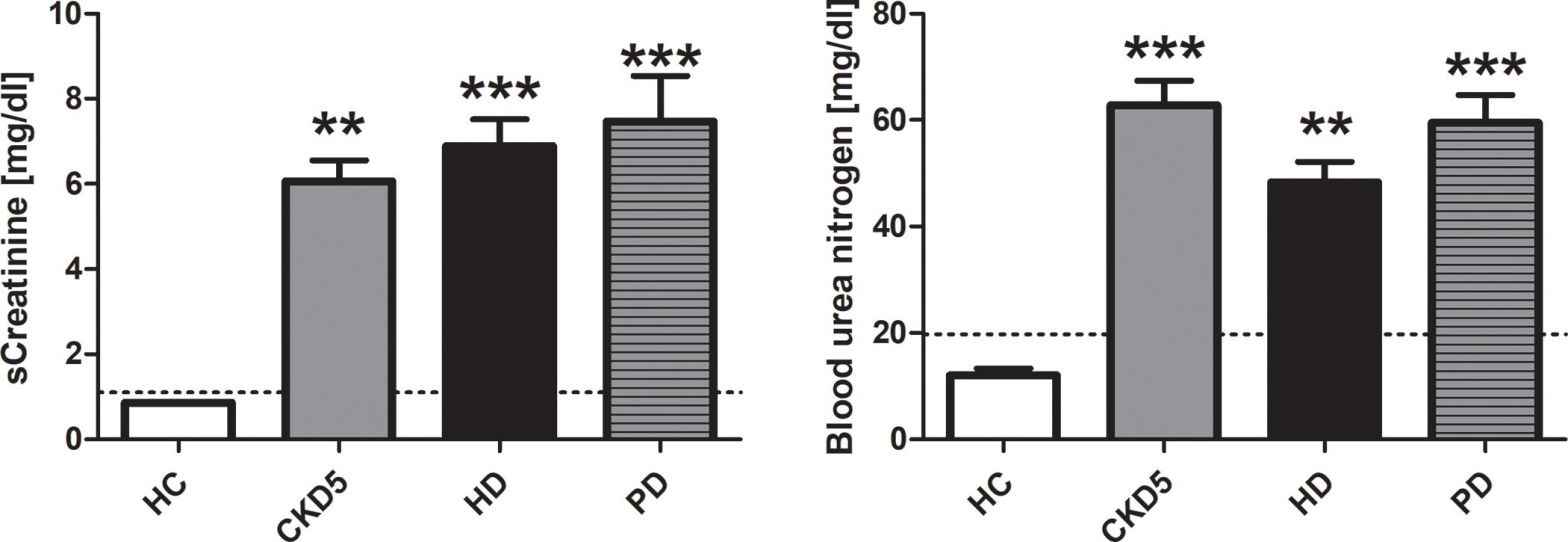
Creatinine and blood urea nitrogen. Values are given as mean ± standard error of the mean for each group. Dotted lines mark upper normal range. **p < 0.01 vs. HC; ***p < 0.001 vs. HC. *HC* healthy control, *CKD5* patients with chronic kidney disease stage G5, *HD* patients on hemodialysis, *PD* patients on peritoneal dialysis.

Serum creatinine (sCrea) and blood urea nitrogen (BUN) were within the normal range in all HC. sCrea and BUN were significantly increased in all other groups compared to HC. sCrea was highest in patients with PD (7.46 ± 1.08 mg/dl), while patients with CKD5 showed highest BUN levels (Fig 1).

### Blood count

Blood count included measurement of leukocytes, erythrocytes, hemoglobin, hematocrit and platelets.

Leukocytes were within the normal range in all HC. Leukocytes were not significantly different in all other groups compared to HC, but one patient with HD and two patients each with CKD5 and PD showed elevated leukocyte levels.

Erythrocytes and hematocrit were within the normal range in all HC, except one who showed a slight anemia by means of hemoglobin and hematocrit. Erythrocytes, hemoglobin and hematocrit were significantly decreased in all patients with end-stage renal disease compared to HC (each p < 0.01). 5 patients with HD and one patient each with CKD5 and PD showed a hemoglobin below 10 g/dl (8.6–9.9 g/dl).

Platelets were within the normal range (180–380 /nl) in all HC. Platelets were not significantly different in all other groups compared to HC, but 12 of 40 patients with end-stage renal disease showed decreased platelets (minimum 99 /nl; median 149 /nl).

### International normalized ratio, activated partial thrombin time, prothrombin time, fibrinogen

Spontaneous international normalized ratio (INR) was 0.98 ± 0.01 in the whole cohort (minimum 0.91; maximum 1.48) with no differences between groups.

Activated partial thrombin time was within the normal range (24.4–32.4 sec) in all HC. aPTT was not significantly different in all other groups with 10 patients with end-stage renal disease being slightly out of the normal range (minimum 21.4 sec; maximum 33.4 sec).

Prothrombin time (PT) was prolonged in one patient with PD. No differences between groups were detected.

**Fig 2:**
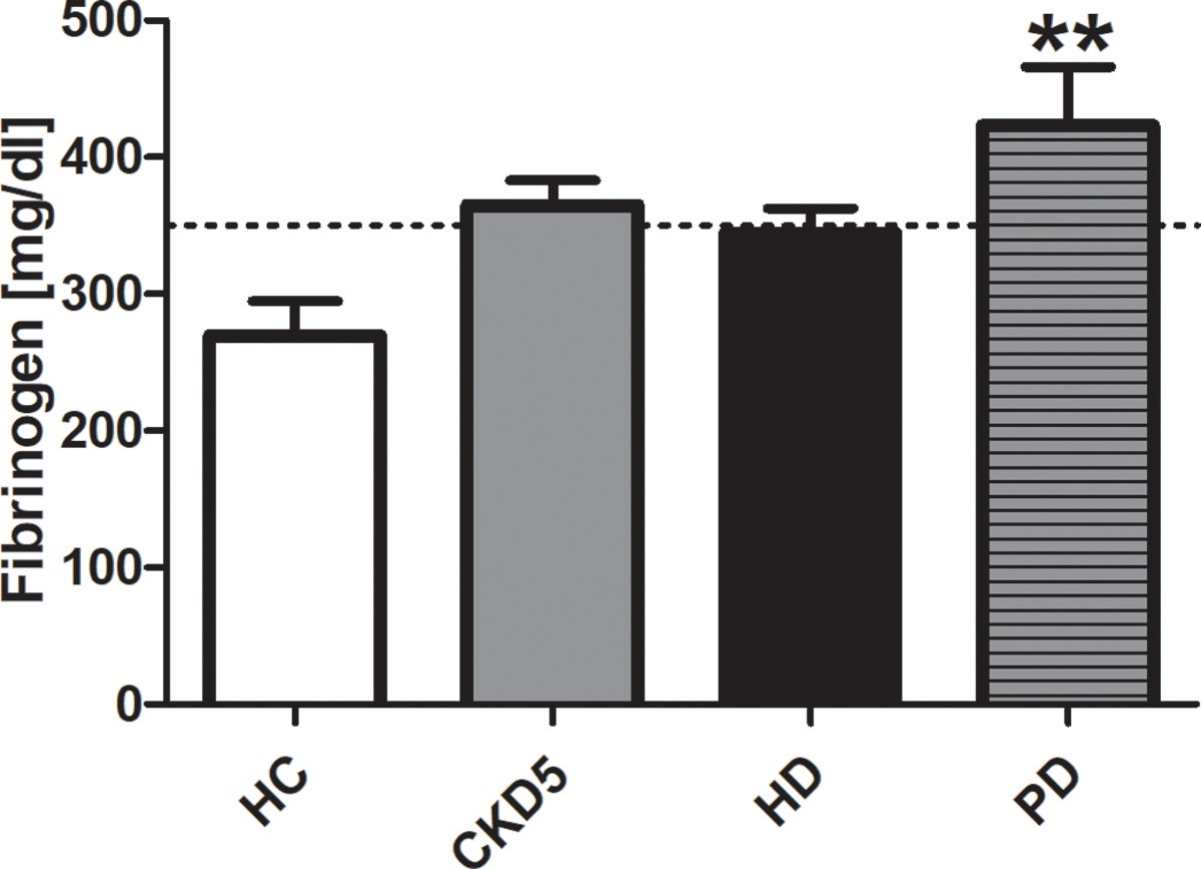
Fibrinogen. Values are given as mean ± standard error of the mean for each group. Dotted line marks upper normal range. **p < 0.01 vs. HC. *HC* healthy control, *CKD5* patients with chronic kidney disease stage G5, *HD* patients on hemodialysis, *PD* patients on peritoneal dialysis.

Fibrinogen was slightly elevated in two HC (269 ± 25 mg/dl). Fibrinogen was significantly increased in patients with PD (Fig 2).

### Platelet function analyzer

Platelet function analyzer was only performed in participants with a hematocrit above 30%.

**Fig 3:**
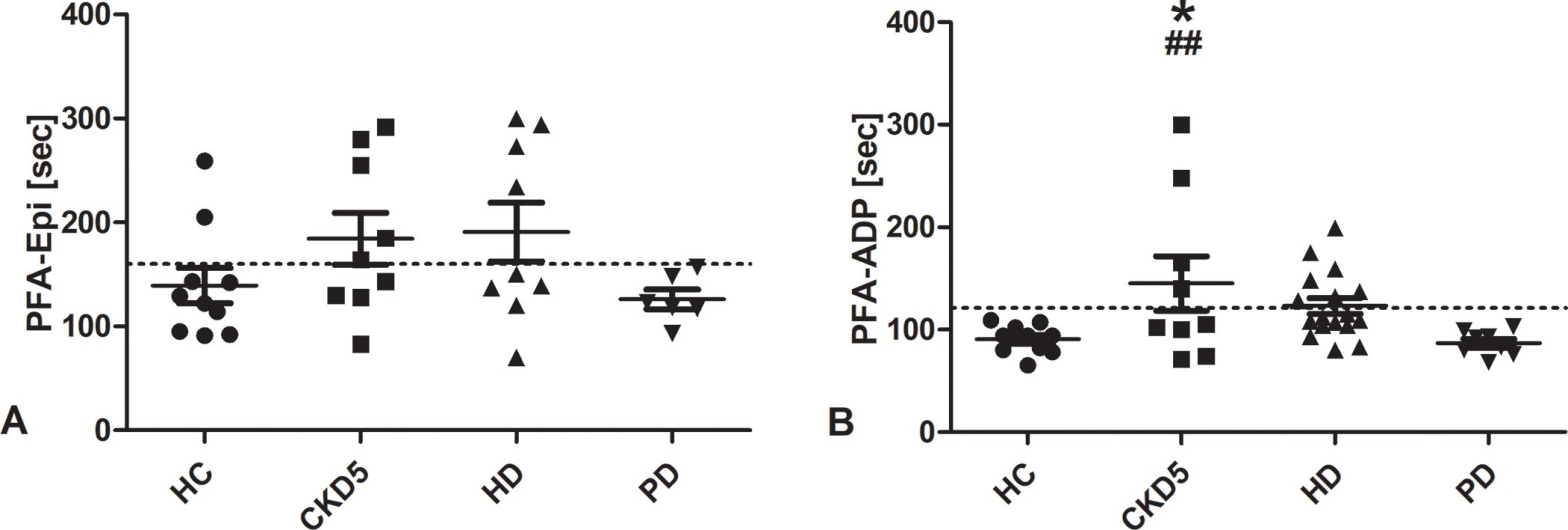
Platelet function analyzer. The platelet function analyzer (PFA) test measured platelet aggregate formation under high shear rates and with addition of either collagen/epinephrine (A) or collagen/adenosine diphosphate (B) as agonists. Lines indicate mean ± standard error of the mean for each group. Dotted lines mark upper normal range. *p < 0.05 vs. HC; ##p < 0.01 vs. PD. *HC* healthy control, *CKD5* patients with chronic kidney disease stage G5, *HD* patients on hemodialysis, *PD* patients on peritoneal dialysis.

PFA-Epi was prolonged in 20% of HC. No PD patient showed a prolonged PFA-Epi, while 56% of patients with CKD5 and 44% of patients with HD did. There was no statistically significant difference between groups (Fig 3A).

PFA-ADP was within the normal range in all HC and patients with PD. It was prolonged in 44% of patients with CKD5 and 41% of patients with HD. For patients with CKD5 the difference was statistically significant compared to HC and patients with PD (Fig 3B).

75–80% of CKD5 and HD patients with a prolonged PFA-Epi also showed a prolonged PFA-ADP.

### Rotational thromboelastometry

Thromboelastometry analysis performed with a ROTEM included INTEM, EXTEM, FIBTEM and APTEM. Parameters assessed included clotting time (CT), clot firmness 10 minutes after CT (A10), maximum clot firmness (MCF) and maximum lysis (ML).

**Fig 4:**
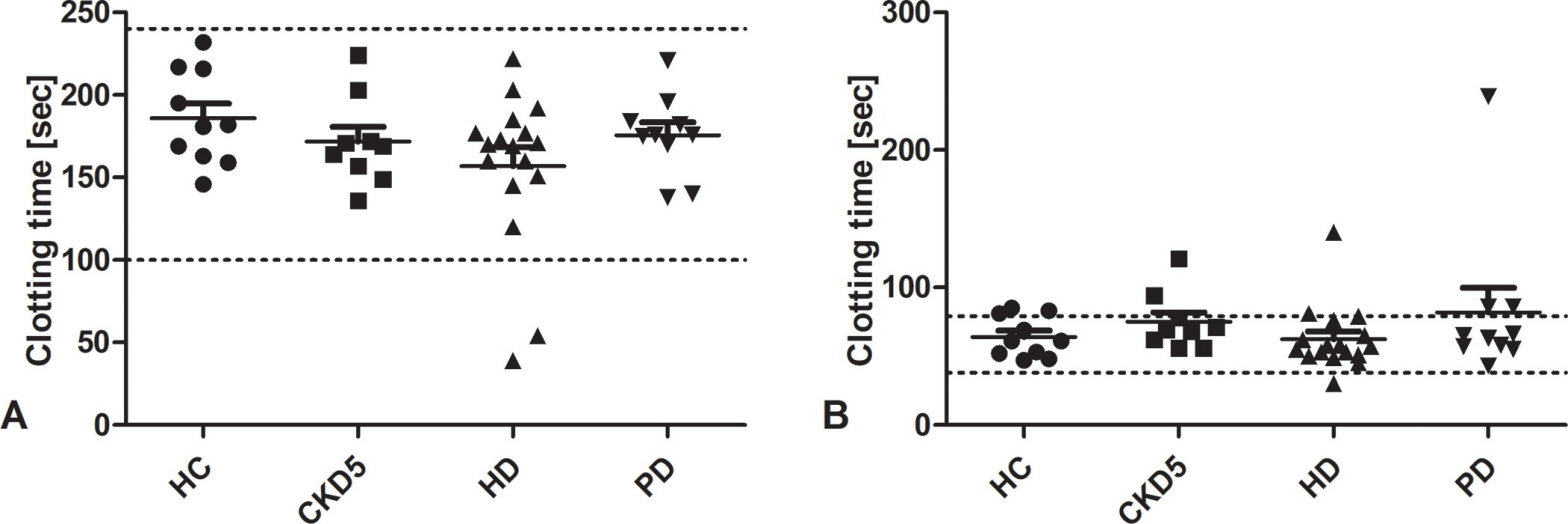
Clotting time measured by rotational thromboelastometry. Clotting time in INTEM (A) corresponds to activated partial thrombin time. Clotting time in INTEM was not prolonged in any patient. Clotting time in EXTEM (B) corresponds to prothrombin time. A strongly prolonged clotting time in EXTEM was found in one patient of every group, except HC with all other measured parameters being unchanged. No differences between groups were detected. Lines indicate mean ± standard error of the mean for each group. Dotted lines indicate normal range. *HC* healthy control, *CKD5* patients with chronic kidney disease stage G5, *HD* patients on hemodialysis, *PD* patients on peritoneal dialysis.

INTEM reflects activation of coagulation by the intrinsic pathway. No evidence for disturbance of coagulation was detected by any of the parameters in any patient. No differences between groups were measured (Fig 4A).

EXTEM reflects activation of coagulation by the extrinsic pathway. A strongly prolonged clotting time was found in one patient of every group, except for HC (Fig 4B). All other measured parameters were insignificantly changed. No differences between groups were detected.

**Fig 5:**
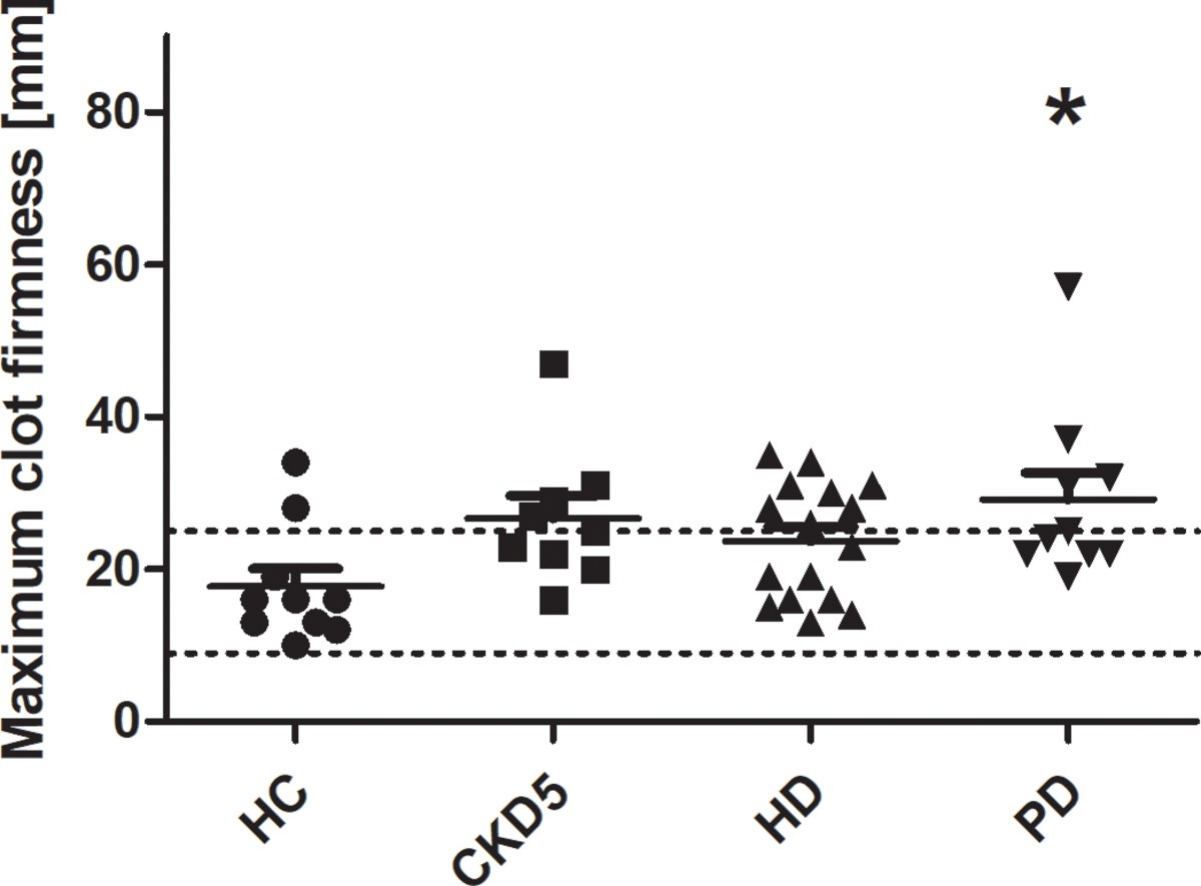
Maximum clot firmness measured by rotational thromboelastometry (FIBTEM). FIBTEM reflects the plasmatic part of clot firmness. Clot firmness tended to be increased in patients with end-stage renal disease becoming significant for PD patients. *p < 0.05 vs. HC. Lines indicate mean ± standard error of the mean for each group. Dotted lines indicate normal range. *HC* healthy control, *CKD5* patients with chronic kidney disease stage G5, *HD* patients on hemodialysis, *PD* patients on peritoneal dialysis.

FIBTEM reflects the plasmatic part of clot firmness. No patient showed reduced clot firmness as detected by FIBTEM. Quite the contrary, maximum clot firmness tended to be increased in patients with end-stage renal disease becoming significant for PD patients (Fig 5). This effect was also shown by an increased A10 (p<0.05 vs. HC).

Hyperfibrinolysis was absent in all patients as measured by comparison of EXTEM and APTEM.

### Multiple electrode aggregometry

**Fig 6:**
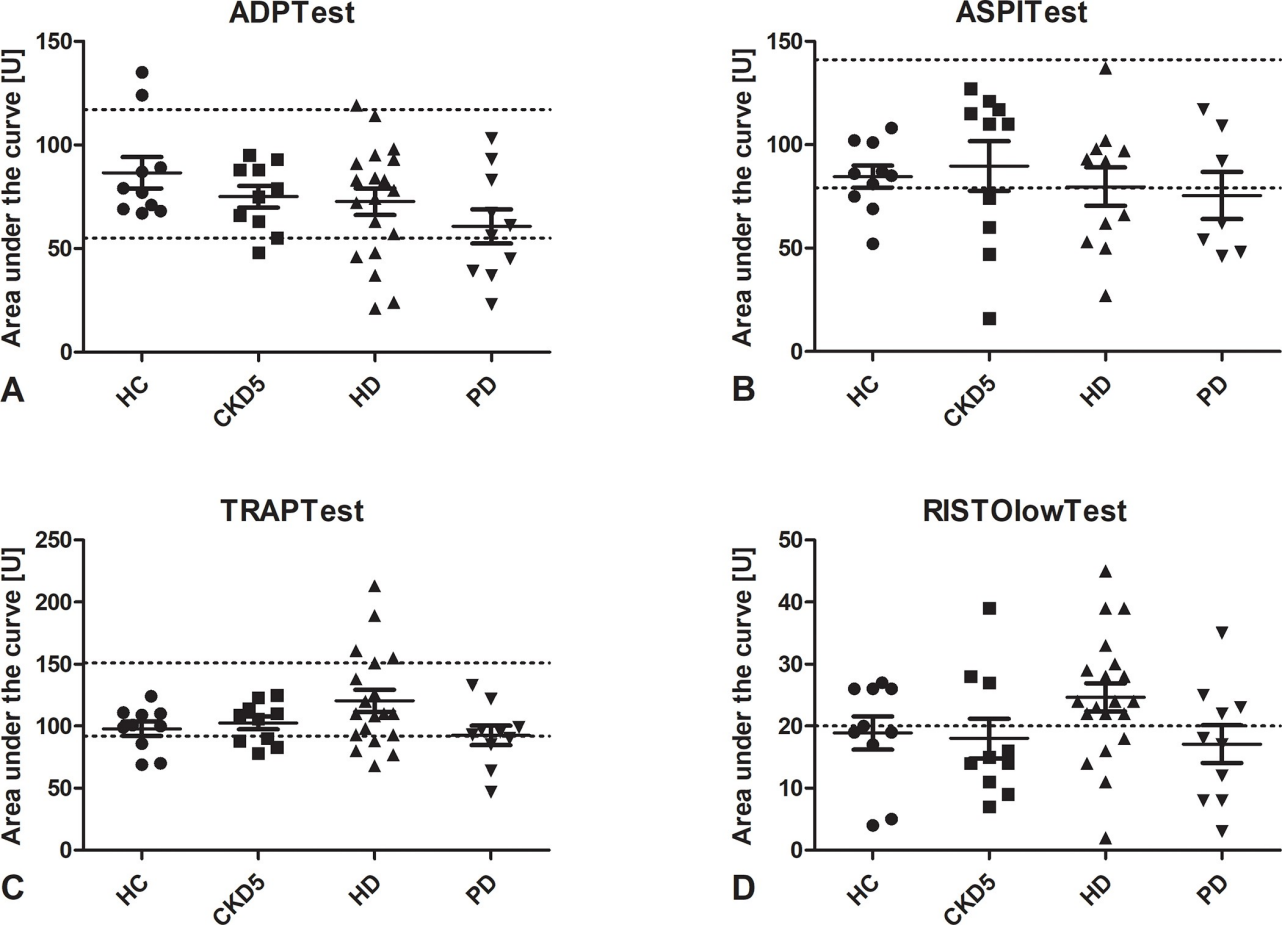
Results of multiple electrode aggregometry. ADPtest (A) reflects function of the P_2_Y_12_ and GpIIb/IIIa receptor. ASPItest (B) reflects function of the platelet cyclooxygenase and the GpIIb/IIIa receptor. TRAPtest (C) reflects function of the protease-activated receptor 1 and the GpIIb/IIIa receptor. RISTOlowTest (D) reflects function of von Willebrand Factor. No significant differences between groups were detected. Lines indicate mean ± standard error of the mean for each group. Dotted lines indicate normal range. *HC* healthy control, *CKD5* patients with chronic kidney disease stage G5, *HD* patients on hemodialysis, *PD* patients on peritoneal dialysis.

Multiple electrode aggregomety performed with a Multiplate included ADP, ASPI, TRAP and Risto low (Fig 6).

The ADPtest reflects platelet function following stimulation of the ADP-receptor. It detects inhibition of the P_2_Y_12_ receptor [11], which can be pharmacologically blocked by clopidogrel, prasugrel and ticlopidin, as well as inhibition or absence of the platelet integrin αIIbβ3 [12, 13]. A reduced area under the curve (AUC) was detected in none of the HC, while 20% of CKD5, 25% of HD and 40% of PD patients showed reduced AUCs. Differences between groups were not statistically significant.

The ASPItest reflects platelet function following stimulation with arachidonic acid. It detects inhibition of the platelet cyclooxygenase [14] as well as inhibition or absence of the platelet integrin αIIbβ3 [12, 15]. A reduced AUC was detected in 20% of HC, while 30% of CKD5, 45% of HD and 57% of PD patients showed reduced AUCs. Differences between groups were not statistically significant.

The TRAPtest reflects platelet function following stimulation with thrombin receptor activating peptide-6 (TRAP-6). It detects function of the protease-activated receptor 1 (PAR-1) and is reduced in case of inhibition or absence of the platelet integrin αIIbβ3 [12, 15]. A reduced AUC was detected in 20% of HC, while 10% of CKD5 and HD, as well as 20% of PD patients showed reduced AUCs. Differences between groups were not statistically significant.

The RISTOlowtest reflects platelet function following induction of agglutination by ristocetin. A strong agglutination response is not expected in the RISTOlowtest, while enhanced aggregation tendency of von Willebrand Factor might result in a higher AUC. An increased AUC was detected in 40% of HC, while 30% of CKD5, 40% of PD and 75% of HD patients (p = 0.06) showed increased AUCs. Differences between groups were not statistically significant.

There was no correlation between the results of the multiple electrode aggregomety and the PFA.

### Thrombin generation assays

**Fig 7:**
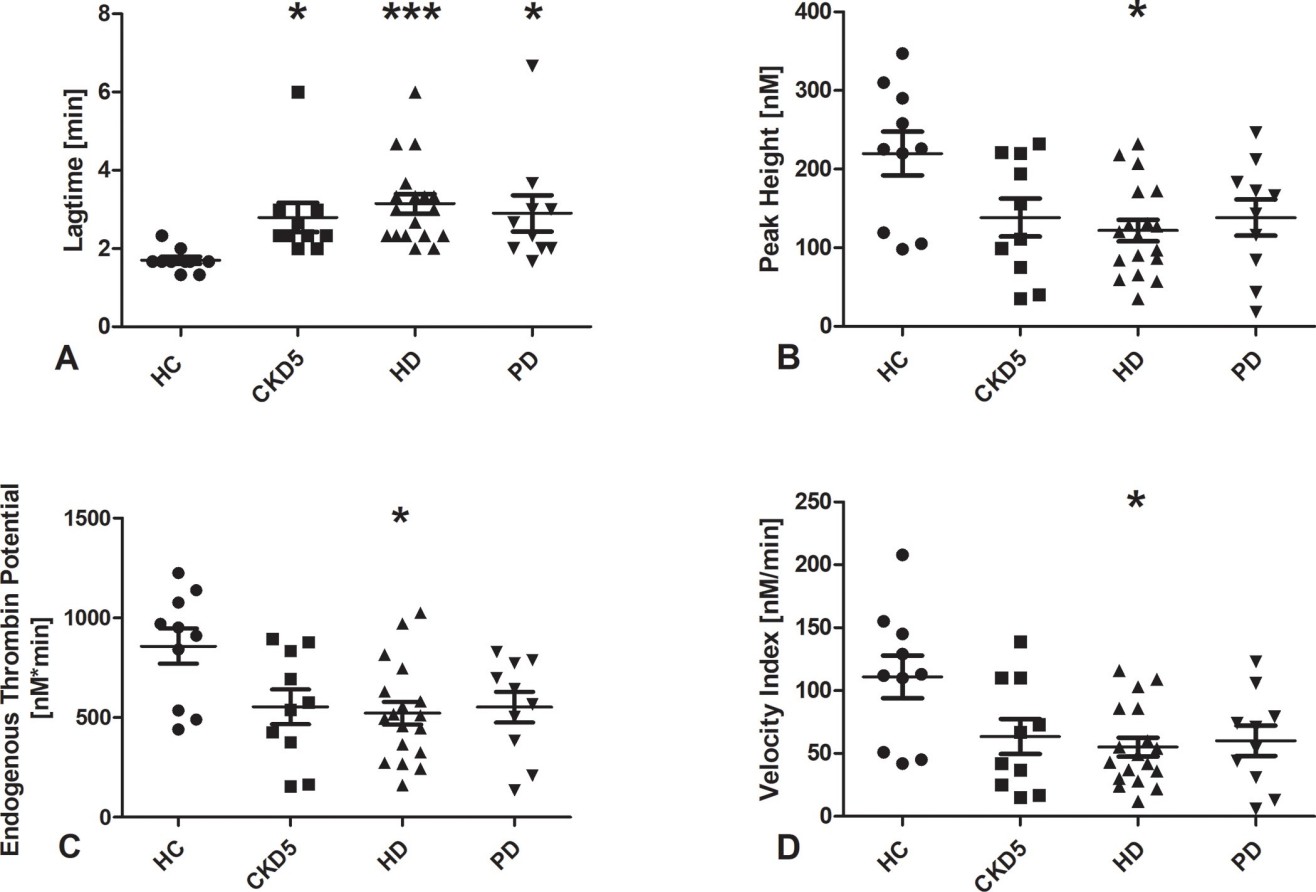
Results of thrombin generation assays. Data are given as medians with interquartile ranges. *p < 0.05 and ***p < 0.001 vs. HC. *HC* healthy control, *CKD5* patients with chronic kidney disease stage G5, *HD* patients on hemodialysis, *PD* patients on peritoneal dialysis.

Results of the TGA are shown in Fig 7.

The TGA reflects the capacity of plasma to generate thrombin after in vitro activation of coagulation.

The lagtime was longer in patients with end-stage kidney disease compared to healthy controls. Peak thrombin, ETP, and velocity index were lower in patients with hemodialysis compared to healthy controls.

### Fibrinolytic potential

**Fig 8:**
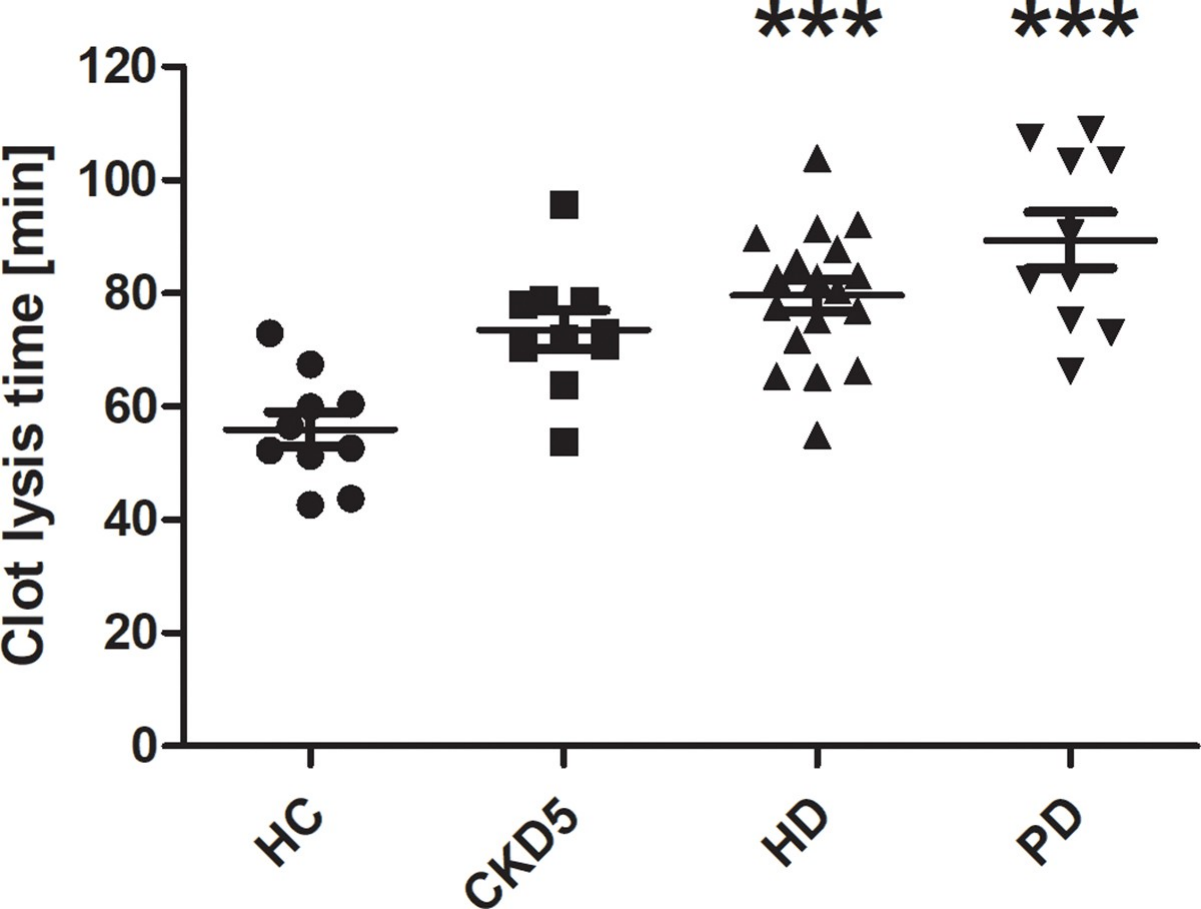
Clot lysis time. Data are given as medians with interquartile ranges. ***p < 0.001 vs. HC. *HC* healthy control, *CKD5* patients with chronic kidney disease stage G5, *HD* patients on hemodialysis, *PD* patients on peritoneal dialysis.

CLT representing fibrinolytic potential was prolonged in patients with HD and PD compared to healthy controls (both p<0.001) indicating a hypofibrinolytic state (Fig 8).

## Discussion

Results of the present study indicate a clopidogrel-like platelet dysfunction in patients with CKD5 and patients with HD treatment becoming concrete in a prolonged PFA-ADP test in patients with CKD5 compared to HC. Multiple electrode aggregometry confirmed a complex platelet defect, although ristocetin-induced agglutination was enhanced in a proportion of patients. Besides platelet dysfunction, thrombin generating potential seems to be decreased in all patients becoming significant for patients on HD.

On the contrary, clot lysis time which is highly significantly prolonged in patients with HD and PD indicates a hypofibrinolytic state. In addition, patients on PD have elevated fibrinogen levels and enhanced whole blood clot formation.

An increased bleeding risk has been described for patients with end-stage renal disease. This bleeding risk was besides anemia and drugs attributed to insufficient platelet function and disturbed interaction between platelets and the vessel walls [2]. On the other hand, end-stage renal disease is also associated with an increased risk for venous thromboembolism [16] and dialysis patients shown an increased risk of pulmonary embolism compared to general population [17] which might even be worse in HD patients than in patients treated by PD [18].

Uremic toxins influence platelet function [19], however BUN levels as a marker for uremic toxins were not linked to any hemostatic alteration in our study being in line with Remuzzi et al. who could not show a correlation between bleeding time and uremic metabolites [20]. Anemia has been described as an important factor for the development of bleeding disorders as hemoglobin is a scavenger of NO and erythrocyte flow leads to localization of platelets along the endothelium encouraging platelet-vessel wall interaction [21, 22]. However, current KDIGO guidelines recommend to only use erythropoietin-stimulating agents for treatment of renal anemia in patients with a hemoglobin ≤ 10 g/dl as a further increase in hemoglobin has shown to provide more harm than benefit [23]. Seven patients showed a hemoglobin below 10 g/dl with none of those having a short-term history of acute bleeding or being symptomatic.

Increased levels of fibrinogen in patients with chronic kidney disease as part of the pro-inflammatory state [24, 25] have been described before. Elevated fibrinogen levels in patients treated with PD [26] might be due long-term peritoneal exposure to especially glucose-based dialysate creating an pro-inflammatory environment exceeding that of HD patients. Increased fibrinogen levels in PD patients were reflect by FIBTEM results of rotational thromboelastometry and may have contributed to a hypofibrinolytic state using a test clearly related to thrombotic disease [27]. No patient in our cohort had switched from HD to PD due to vessel access problems. A more prothrombotic state of PD patients compared to HD patients has been described before [26].

PFA-Epi was prolonged in 56% of patients with CKD5 and 44% of patients with HD, PFA-ADP was prolonged in 44% of patients with CKD5 and 41% of patients with HD. Although results were only significant compared to HC for PFA-ADP in CKD5 patients tests indicate a platelet disorder. As platelets are involved in primary hemostasis this might cause problems in case of acute (traumatic) bleedings from cannulations sides, the gastrointestinal tract or following (kidney) biopsies or surgery. The differences between PFA and multiple electrode aggregometry, which showed a tendency for a higher AUC in RISTOlow indicating an enhanced aggregation tendency at least for HD patients, are not per se surprising as PFA measures adhesion and aggregation under flow, whereas multiple electrode aggregometry is a test of aggregation only. Therefore, the results are in line with Zwaginga et al., who found no platelet defect in suspension aggregation, but platelet defects in experimental flow models with uremic blood [28]. In addition, it was reported previously that high levels of the platelet adhesive protein von Willebrand factor compensate for the platelet function defect in patients with renal disease [29], which are in line with our current results on enhanced platelet agglutination by ristocetin, which is dependent on von Willebrand factor. The differences between HD and PD on platelets are profound, with more ex vivo platelet activation (and thrombocytopenia) and dilution induced in the HD circuit. Every available platelet test measures a different aspect of platelet function, and correlation between tests is very poor.

Although thrombin generating potential was decreased to some degree in all patients with end-stage kidney disease, which was shown before [10], a hypercoagulable state was found especially for PD patients going along with a generally increased risk for venous thromboembolism. Our results are in line with Nieuwenhuijs-Moeke at al. who showed an hypercoagulable state in preemptively and non-preemptively transplanted patients tested during surgery compared to living kidney donors [10]. Detection of platelet function disorders stays rather vague and inhomogeneous, but explains bleeding complications following interventions. The general concept that renal disease patients are not only bleeders is supported by both clinical and laboratory evidence.

There are some limitations to our study. Due to limited cohort size the impact of the underlying diseases on hemostasis could not be addressed. None of the patients included had been diagnosed with a coagulation disorder. Medication was only considered with respect to inhibitors of platelet aggregation and systemic anticoagulation. None of the patients included received antibiotics.

A power calculation was not performed as we did not have data on the variation of the various hemostatic tests performed in the particular patient groups. As the number of measurements was limited, we did not include tests depicting interaction of platelets and vessel wall such as von Willebrand factor.

## Conclusions

In conclusion, our results indicate that patients with end-stage renal disease have clear hypercoagulable features, which is not in line with the clinical concept of patients with end-stage renal disease being bleeders. However, end-stage renal disease seems to be associated with platelet dysfunction which disables primary hemostasis in cases of acute bleeding. At this time, it is impossible to conceive the global hemostatic state of an individual patient in a feasible and accepted test model.

## Supporting information

S1 File Raw data. Detailed results of all experiments performed.
(XML)
